# Membrane potential and cancer progression

**DOI:** 10.3389/fphys.2013.00185

**Published:** 2013-07-17

**Authors:** Ming Yang, William J. Brackenbury

**Affiliations:** Department of Biology, University of YorkYork, UK

**Keywords:** cancer, cell cycle, differentiation, ion channel, membrane potential, migration, proliferation, stem cell

## Abstract

Membrane potential (*V_m_*), the voltage across the plasma membrane, arises because of the presence of different ion channels/transporters with specific ion selectivity and permeability. *V_m_* is a key biophysical signal in non-excitable cells, modulating important cellular activities, such as proliferation and differentiation. Therefore, the multiplicities of various ion channels/transporters expressed on different cells are finely tuned in order to regulate the *V_m_*. It is well-established that cancer cells possess distinct bioelectrical properties. Notably, electrophysiological analyses in many cancer cell types have revealed a depolarized *V_m_* that favors cell proliferation. Ion channels/transporters control cell volume and migration, and emerging data also suggest that the level of *V_m_* has functional roles in cancer cell migration. In addition, hyperpolarization is necessary for stem cell differentiation. For example, both osteogenesis and adipogenesis are hindered in human mesenchymal stem cells (hMSCs) under depolarizing conditions. Therefore, in the context of cancer, membrane depolarization might be important for the emergence and maintenance of cancer stem cells (CSCs), giving rise to sustained tumor growth. This review aims to provide a broad understanding of the *V_m_* as a bioelectrical signal in cancer cells by examining several key types of ion channels that contribute to its regulation. The mechanisms by which *V_m_* regulates cancer cell proliferation, migration, and differentiation will be discussed. In the long term, *V_m_* might be a valuable clinical marker for tumor detection with prognostic value, and could even be artificially modified in order to inhibit tumor growth and metastasis.

## Introduction

The presence of various ion channels and transporters at the plasma membrane provides different permeability to distinct ions, such as Na^+^, K^+^, Ca^2+^, and Cl^−^. Due to the unequal distribution of these ions, a voltage difference exists between the cytoplasm and the extracellular environment, which is known as the membrane potential (*V_m_*). *V_m_* is expressed relative to the extracellular environment. A cell is depolarized when the *V_m_* is relatively less negative, whereas a hyperpolarized cell possesses a more negative *V_m_*. *V_m_* changes because of alterations in the conductance of one or more types of ion. The Goldman–Hodgkin–Katz equation shows that the *V_m_* depends on the permeability (P) and both the intracellular and extracellular concentrations of major ions (Goldman, 1943; Hodgkin and Katz, 1949):

$$V_{m}=\frac{RT}{F}\text{ln}\left(\frac{P_{{\text{Na}}^{+}}{\left[{\text{Na}}^{+}\right]}_{o}+P_{{\text{K}}^{+}}{\left[{\text{K}}^{+}\right]}_{o}+P_{{\text{Cl}}^{-}}{\left[{\text{Cl}}^{-}\right]}_{o}}{P_{{\text{Na}}^{+}}{\left[{\text{Na}}^{+}\right]}_{i}+P_{{\text{K}}^{+}}{\left[{\text{K}}^{+}\right]}_{i}+P_{{\text{Cl}}^{-}}{\left[{\text{Cl}}^{-}\right]}_{i}}\right)$$

where *R* is the ideal gas constant, *T* the temperature, and *F* the Faraday constant. In addition, intercellular communications (e.g., gap junction connections) are also able to influence *V_m_* (Hulser and Lauterwasser, 1982; Levin, 2007a). In excitable cells, such as neurons and muscle fibers (Nakajima and Horn, 1967; Bean, 2007), changes in *V_m_* underlie the action potential (AP) waveform. APs fire in response to a depolarization that exceeds a threshold value. Fine-tuning of APs is tightly regulated by the activities of several key ion channels and transporters, including voltage-gated Na^+^ channels (VGSCs), voltage-gated K^+^ channels (K_*v*_), and the Na^+^/K^+^-ATPase (Caldwell and Keynes, 1957; Hille, 1992).

Emerging evidence suggests that the *V_m_* also plays important functional roles in non-excitable cells. In the late 1960's, while studying mitotic activities in sarcoma cells, Clarence D. Cone Jr. reported that *V_m_* underwent hyperpolarization before entering M phase, and suggested that the level of *V_m_* correlated with cell cycle progression (Cone, 1969). He subsequently showed that membrane hyperpolarization reversibly blocked DNA synthesis and mitosis (Cone, 1970). He later generalized existing data at that time and postulated that the *V_m_* level was correlated with the level of differentiation. For example, terminally differentiated cells (e.g., fibroblasts and epithelium) possess hyperpolarized *V_m_* (Cone, 1971). Since then, changes in *V_m_*, representing the long-term, slowly changing bioelectric gradient in non-excitable cells (Lobikin et al., 2012), have been shown to control critical cell functions including proliferation, migration, and differentiation (Binggeli and Weinstein, 1986; Schwab et al., 2007; Blackiston et al., 2009; Sundelacruz et al., 2009). Recently, studies have also demonstrated that *V_m_* is able to, directly or indirectly, control wound healing (Nuccitelli, 2003a,b; McCaig et al., 2009), left-right patterning (Adams et al., 2006), development (Nuccitelli, 2003a; Adams, 2008), and regeneration (Levin, 2007b, 2009). Therefore, given the increasing evidence showing that ion channels/transporters functionally participate in cancer progression (Kunzelmann, 2005; Fiske et al., 2006; Stuhmer et al., 2006; Prevarskaya et al., 2010; Becchetti, 2011; Brackenbury, 2012), it is not surprising that *V_m_* has been implicated in cancer development, since *V_m_* is itself determined by the combined activities of ion channels/transporters at the cell membrane. This article aims to summarize current understanding of the *V_m_* as a bioelectric regulator in cancer, and examines the therapeutic potential of *V_m_* for tumor detection and treatment.

## Cancer cells possess depolarized *V_m_*

Cone's theory proposing the general correlation between proliferation and *V_m_* (Cone, 1971) was supported by several previous studies which demonstrated significant *V_m_* depolarization during malignant transformation of normal cells (Tokuoka and Morioka, 1957; Johnstone, 1959). Direct *in vitro* and *in vivo* comparisons of *V_m_* levels between normal and cancerous breast cells (Marino et al., 1994), hepatocytes and hepatocellular carcinoma cells (Binggeli and Cameron, 1980; Stevenson et al., 1989), normal and neoplastic adrenocortical tissues (Lymangrover et al., 1975), normal embryonic fibroblasts and fibrosarcoma (Binggeli and Weinstein, 1985), benign and cancerous skin cells (Melczer and Kiss, 1957; Woodrough et al., 1975), and between normal and cancerous ovarian tissue (Redmann et al., 1972) showed that cancer cells tended to be more depolarized than their normal counterparts. In addition, the intracellular Na^+^ level is markedly higher in tumors compared to non-cancerous tissues, whereas the K^+^ level remains more stable (Smith et al., 1978; Cameron et al., 1980; Sparks et al., 1983). A similar scenario occurs in fast proliferating Chinese hamster ovary (CHO) and 3T3 cells (Cone and Tongier, 1973). Thus, an increased intracellular Na^+^ concentration could be a determinant of a depolarized phenotype in rapidly cycling cancer cells.

**Figure 1:**
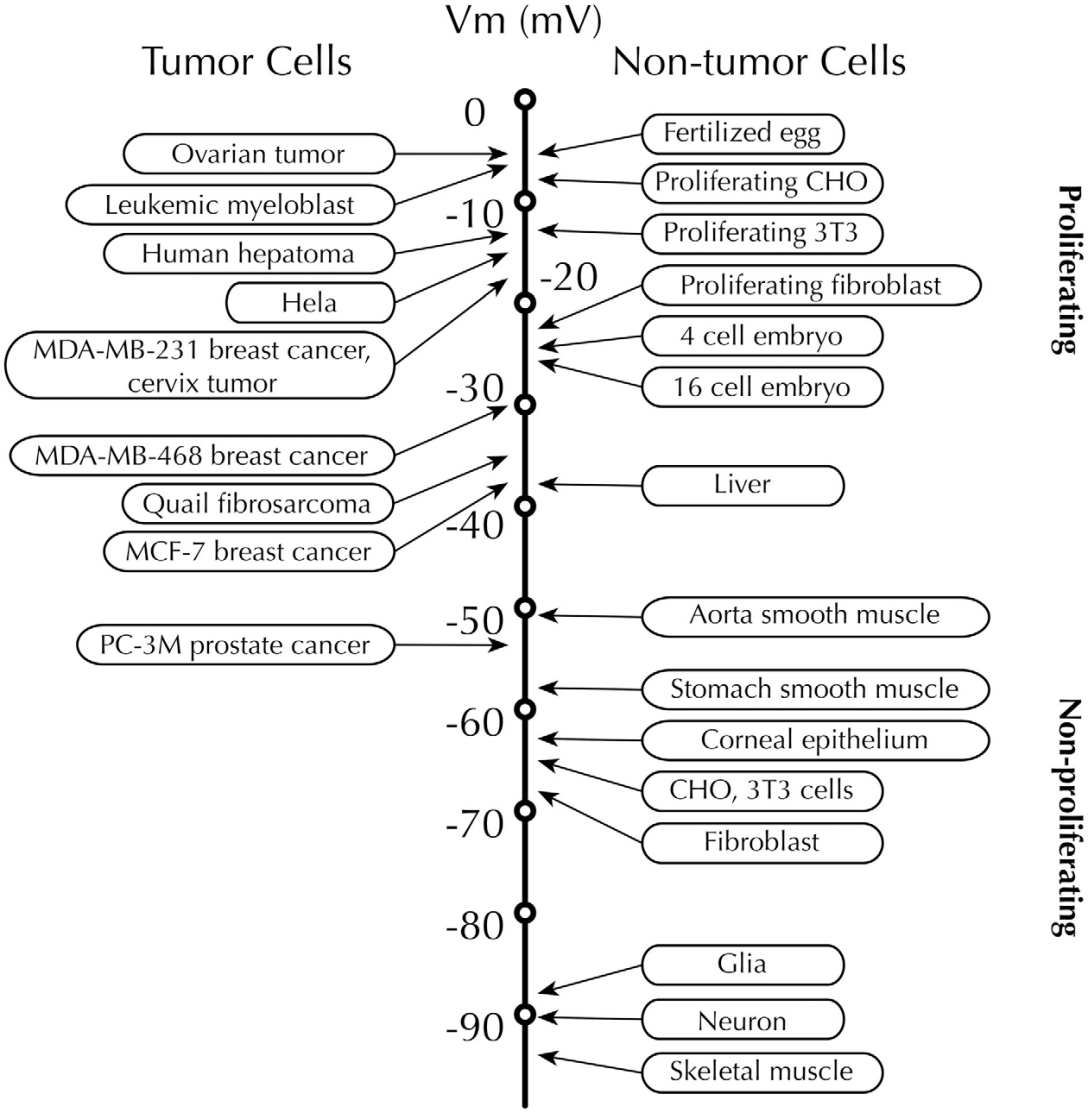
**Membrane potential (*V_m_*) scale**. Rapidly proliferating cancer cells possess depolarized *V_m_*, while the *V_m_* of quiescent cells is generally more negative. Proliferative somatic cells are also depolarized, suggesting that *V_m_* is functionally instructive in cell development (Levin, 2007b). Scale adapted from Binggeli and Weinstein (1986), with additional data from Fraser et al. (2005); Mycielska et al. (2005); Yang et al. (2012).

Recordings from rodent and human tissues have revealed that proliferative cells, especially rapidly proliferating tumor cells, displayed depolarized *V_m_*, whereas non-proliferating, terminally differentiated somatic cells, such as muscle cells and neurons, are characterized by their hyperpolarized *V_m_* (Figure 1) [reviewed in Binggeli and Weinstein (1986)]. Given these findings, is *V_m_* merely an epiphenomenon, which only indicates the outcome of the activities of various ion channels and transporters, or is it is actually a functional instructor that is capable of promoting tumorigenesis? A similar question had been posed 50 years ago soon after Cone revealed the relationship between mitotic activity and *V_m_* level (Cone and Tongier, 1971). For example, depolarization can initiate mitosis in CHO cells and mouse spleen lymphocytes (Cone and Tongier, 1971; Kiefer et al., 1980). By contrast, hyperpolarized *V_m_* immediately precedes mitotic arrest (Cone and Tongier, 1973). More recently, *in vivo* evidence shows that membrane depolarization itself, regardless of the types of ions and ion channel/transporter proteins, is able to bring cancerous transformation (i.e., increased proliferation, change in morphology and abnormal angiogenesis) in *Xenopus laevis* embryos (Lobikin et al., 2012).

Hanahan and Weinberg proposed 10 hallmarks of cancer, including sustaining proliferative signaling, activating invasion and metastasis, and angiogenesis (Hanahan and Weinberg, 2011). The following sections review the prevailing evidence that implicates *V_m_* in several of these processes.

## *V_m_* and cancer cell proliferation

**Figure 2:**
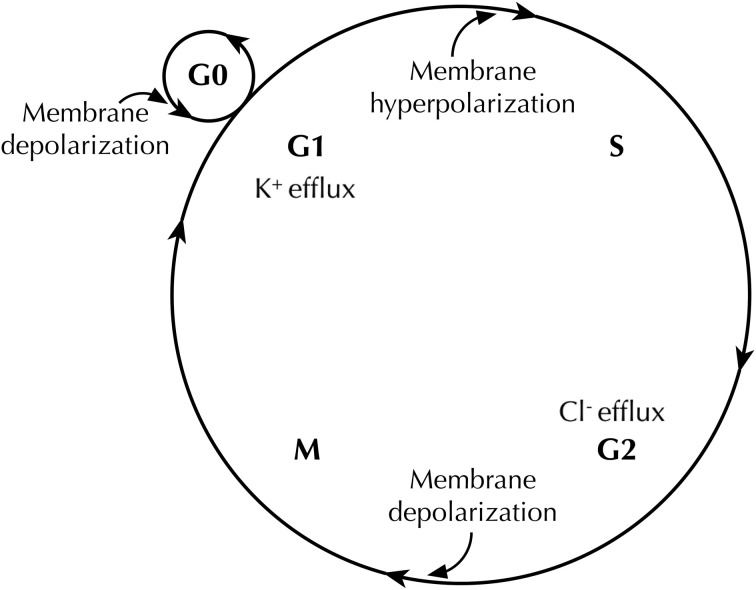
**Membrane potential (*V_m_*) changes during the cell cycle**. *V_m_* undergoes hyperpolarization at G_1_/S border, by virtue of K^+^ efflux through various K^+^ channels. Before cells enter M phase, increased Cl^−^ efflux accompanies *V_m_* depolarization. Quiescent cells at G_0_ stage show mitotic activities after *V_m_* depolarization (Cone and Cone, 1976).

In general, in both highly proliferative tumor and non-tumor cells, depolarization is believed to serve as a signal that could initiate mitosis and DNA synthesis (Orr et al., 1972; Binggeli and Weinstein, 1986). Artificially altering *V_m_* by modulating the extracellular ionic constitution or applying the Na^+^/K^+^-ATPase inhibitor ouabain revealed interesting results: First, hyperpolarizing CHO cells to −45 mV started to induce mitotic arrest and cell division was fully blocked at −75 mV. The cell cycle was resumed by depolarizing the cells to −10 mV (Cone, 1971). Secondly, quiescent (G_0_) mature chick spinal cord neurons showed mitotic activity after depolarization (Cone and Cone, 1976) (Figure 2). Recently, artificial control of *V_m_* was accomplished in *Xenopus laevis* embryos by expressing glycine-gated Cl^−^ channels and applying the activator ivermectin. Depolarization (caused by lowering the Cl^−^ concentration in the extracellular medium, which caused Cl^−^ efflux) was found to be directly responsible for malignant proliferation. This proliferation was ion and ion channel non-specific, because (1) the phenotype caused by depolarization could be rescued by expressing a hyperpolarizing channel gene, and (2) the malignant phenotype could be induced or suppressed simply by adjusting extracellular Cl^−^ concentration, as predicted by Goldman–Hodgkin–Katz equation (Lobikin et al., 2012). Therefore, the depolarized *V_m_* frequently found in cancerous cell types could be regarded as a “sustaining proliferative signal” that instructs cells to rapidly advance in the cell cycle.

An additional layer of complexity in this model is that the *V_m_* fluctuates during cell cycle progression, and follows a multi-step and rhythmic pattern (Wonderlin and Strobl, 1996; Blackiston et al., 2009) (Figure 2). A number of studies suggest that membrane hyperpolarization at the G_1_/S checkpoint is generally required for S phase initiation. For example, depolarizing the cell membrane halts G_1_/S progression in glia (Canady et al., 1990), Schwann cells (Wilson and Chiu, 1993), lymphocytes (Price et al., 1989; Freedman et al., 1992; Wang et al., 1992), V79 Chinese hamster lung cells (Sachs et al., 1974), C1300 mouse neuroblastoma cells (Boonstra et al., 1981), and MCF-7 human breast cancer cells (Wonderlin et al., 1995). The *V_m_* then appears to remain relatively hyperpolarized through S phase in some cell types (Sachs et al., 1974; Boonstra et al., 1981; Strobl et al., 1995; Wonderlin et al., 1995), but is more depolarized in others (Arcangeli et al., 1995; Macfarlane and Sontheimer, 2000). The G_2_/M transition exhibits a depolarized *V_m_* (Sachs et al., 1974; Boonstra et al., 1981; Blackiston et al., 2009), although it is not known whether or not this depolarization is a prerequisite for progression. In fact, the exact *V_m_* thresholds for driving progression appear to depend heavily on cell type, the state of differentiation, and the density of cell monolayer in culture (Cone and Tongier, 1973; Blackiston et al., 2009).

Importantly, the fluctuation of *V_m_* levels across the cell cycle does not necessarily contradict the observation that depolarized *V_m_* could be a hallmark of cancer cells. The mean *V_m_* values in cancer cells are consistently depolarized relative to most normal somatic cell types (Figure 1). For example, MCF-7 cells arrested at G_1_ phase have a *V_m_* of −9 mV and hyperpolarize to ~−30 mV in the S phase (Wonderlin et al., 1995). Both these values are more depolarized than normal breast cells, e.g., the mean *V_m_* of unsynchronized MCF-10A cells is between −40 and −58 mV (Marino et al., 1994; Wonderlin et al., 1995; Fraser et al., 2005).

Evidence suggests that the fluctuation in K^+^ concentration plays a significant contribution to changes in *V_m_* during the cell cycle. For example, in neuroblastoma and Ehrlich ascites cells, there is a transient decrease in K^+^ efflux before entering the G_2_ phase, a relatively high level of K^+^ efflux during the M phase (Mills and Tupper, 1976; Boonstra et al., 1981). Given the diversity of K^+^ channel types (Hille, 1992; Miller, 2000; Wang, 2004), their relative contributions to the *V_m_* and *V_m_*-dependent cell cycle progression is probably context-dependent and highly complex. For example, inhibition of cell proliferation with K^+^ channel inhibitors does not correlate with changes in the *V_m_* in rat C6 glioma cells (Rouzaire-Dubois et al., 2000). In addition, the *V_m_* is likely to be determined by the collective activities of a variety of ions/channels/transporters, which may exhibit reciprocal interactions and form a large and complex network responsible for *V_m_* regulation and its downstream effects.

## Ion channel-dependent regulation of proliferation and *V_m_*

**Figure 3:**
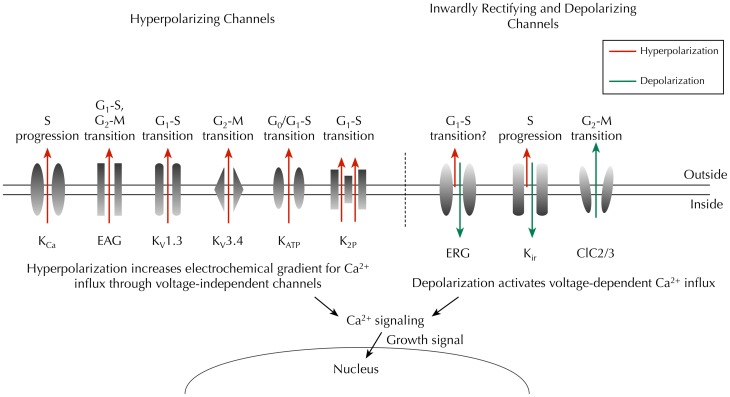
**Key ion channels that regulate *V_m_* and cell cycle progression in cancer**. Hyperpolarizing channels (outward I_*K*_, red) would increase the driving force for Ca^2+^ influx through voltage-independent channels, whereas inwardly rectifying K^+^ channels (predominantly inward I_*K*_, green) and chloride channels (outward Cl^−^, green) would depolarize the *V_m_*, thus enabling activation of voltage-dependent Ca^2+^ influx (Schwab et al., 2012). Time- and domain-dependent Ca^2+^ signaling is then proposed to activate pathways that promote cell cycle progression and proliferation. Abbreviations: K_*Ca*_, Ca^2+^-activated K^+^ channel; EAG, *ether à go-go* channel; K_*v*_, voltage-gated K^+^ channel; K_*ATP*_, ATP-sensitive K^+^ channel; K_*2P*_, two-pore domain K^+^ channel; ERG, EAG-related gene K^+^ channel; K_*ir*_, classic inward-rectifier K^+^ channel; ClC2/3, chloride 2/3 channel.

Numerous studies have shown that pharmacological or genetic block of K_*v*_ channels reduces proliferation of cancer cells (e.g., Fraser et al., 2000; Ouadid-Ahidouch et al., 2000; Abdul and Hoosein, 2002; Chang et al., 2003; Menendez et al., 2010). Increasing evidence suggests that *Ether à go-go* (EAG) K^+^ channels may serve as biomarkers for cancer (Ouadid-Ahidouch et al., 2001; Farias et al., 2004; Pardo et al., 2005; Hemmerlein et al., 2006; Ousingsawat et al., 2007; Ortiz et al., 2011; Rodriguez-Rasgado et al., 2012). Inhibition of EAG channel expression reduces proliferation in several cancer cell lines, whereas implantation of CHO cells over-expressing EAG channels in mice induces tumors (Pardo et al., 1999). In synchronized SH-SY5Y cells, human I_*EAG*_ is reduced to less than 5% in G_1_ phase, compared to unsynchronized controls, suggesting that the activity of EAG channels is cell cycle-dependent (Meyer and Heinemann, 1998). Indeed, in MCF-7 cells, inhibiting EAG channels with astemizole increases the proportion of cells in G_1_ phase and reduces the proportion in S phase (Borowiec et al., 2007). In contrast, activation of hEAG channels is responsible for hyperpolarization at late G_1_ before the cells enter the S phase (Ouadid-Ahidouch et al., 2001). Interestingly, the hyperpolarization is accompanied by increased Ca^2+^-activated K^+^ (K_*Ca*_) channel currents (Ouadid-Ahidouch et al., 2001), which might result from the elevated intracellular Ca^2+^ due to the increased electrochemical gradient (Figure 3) (Nilius and Wohlrab, 1992; Ouadid-Ahidouch and Ahidouch, 2008).

When K_*Ca*_ channels were found in Friend murine erythroleukemia cells, they were thought to be one of the main controllers of the *V_m_* (Arcangeli et al., 1987). K_*Ca*_ channels have been found since in glioma (Liu et al., 2002), prostate cancer (Gessner et al., 2005), breast cancer (Haren et al., 2010), and the CD133^+^ subpopulation of SH-SY5Y cells (Park et al., 2010). Inhibiting K_*Ca*_ channels with iberiotoxin arrests D54-MG glioma cells in S phase, and leads to apoptosis (Weaver et al., 2004). Thus, the functional contribution of K_*Ca*_ channels to cell cycle regulation appears to be distinct from K_*v*_ channels. In addition, in MCF-7 cells, inhibition of ATP-sensitive K^+^ (K_*ATP*_) channels reversibly arrests cells in the G_0_/G_1_ phase (Woodfork et al., 1995). The two-pore domain K^+^ channel, TREK1, increases proliferation of PC-3 and LNCaP prostate cancer cells (Voloshyna et al., 2008). In CHO cells, overexpression of TREK1 increases the number of cells in S phase, and reduces the number of cells at G_0_/G_1_ phase (Voloshyna et al., 2008).

Human EAG-related gene (HERG) K^+^ channels are strongly inwardly rectifying and conduct K^+^ influx when the voltage is more negative than the K^+^ equilibrium potential (Trudeau et al., 1995; Smith et al., 1996). HERG channels are expressed at early developmental stages in the neural crest, central nervous system, dorsal root ganglion (DRG) and skeletal muscle, and are replaced by classic inward rectifier K^+^ current (IK_*ir*_) later in development (Arcangeli et al., 1997; Crociani et al., 2000). HERG channels are upregulated in a number of cancers (Arcangeli, 2005). Moreover, I_*HERG*_ increases tumor cell proliferation (Bianchi et al., 1998; Wang et al., 2002). The activity of I_*HERG*_ itself is cell cycle dependent (Arcangeli et al., 1995), suggesting a complex relationship between I_*HERG*_, *V_m_*, and proliferation. Additional inward rectifier K^+^ (K_*ir*_) channels have been reported in various cancer cell types, and are required for proliferation, including K_*ir*_2.2 (Lee et al., 2010), K_*ir*_3.1, and K_*ir*_3.4 (Plummer et al., 2004; Takanami et al., 2004; Plummer et al., 2005; Wagner et al., 2010). In contrast, overexpression K_*ir*_4.1 in glioma cells hyperpolarizes the *V_m_* and increases the number of cells in quiescent G_0_/G_1_, reducing the proportion in G_2_/M phase (Higashimori and Sontheimer, 2007). Thus, different K_*ir*_ channels may play opposing roles in regulation of *V_m_*/proliferation, as a result of their heterogeneous voltage dependence (Figure 3). Cl^−^ conductance also appears to be linked to the cell cycle and regulate proliferation. For example, in D54-MG cells, Cl^−^ efflux through the outward rectifying ClC3 Cl^−^ channel is significantly increased during M phase (Habela et al., 2008). In addition, the ClC2 channel is expressed in M phase in transfected NRK-49F rat kidney fibroblast cells (Zheng et al., 2002).

The mechanisms underlying ion channel-dependent proliferation of cancer cells have been reviewed in detail elsewhere (Wang, 2004; Ouadid-Ahidouch and Ahidouch, 2008; Prevarskaya et al., 2010). These include possible non-conducting, direct interactions between ion channels and other pro-proliferative signaling mechanisms. For example, coexpression of HERG and tumor necrosis factor receptor 1 (TNFR1) has been found at the cell membrane of SKBR3 and SH-SY5Y cell lines, and HERG appears to recruit TNFR1 to the membrane, therefore enhancing TNF-α-induced cancer cell proliferation (Wang et al., 2002). Alternatively, ion channel-mediated *V_m_* hyperpolarization would increase the electrochemical gradient for Ca^2+^ and therefore elevate the intracellular Ca^2+^ concentration through voltage-independent Ca^2+^ channels, such as transient receptor potential (TRP) channels (Nilius and Wohlrab, 1992; Wang, 2004; Ouadid-Ahidouch and Ahidouch, 2008). Ca^2+^ signaling is functional across the whole cell cycle (Santella et al., 2005). For example, Ca^2+^ is required for G_1_ progression and G_1_/S transition (Hazelton et al., 1979; Choi et al., 2006). In turn, intracellular Ca^2+^ and calmodulin (CaM) can regulate K_*Ca*_ and EAG channels (Khanna et al., 1999; Ziechner et al., 2006; Ouadid-Ahidouch and Ahidouch, 2008). Thus, there may be a reciprocal, auto-regulatory relationship between ion channel activity, *V_m_*, intracellular Ca^2+^ signaling, and proliferation.

In summary, a multiplicity of ion channels (predominantly K^+^-conducting) participates in *V_m_* regulation (both depolarization and hyperpolarization) in cancer cells. In turn, changes in *V_m_* promote transition through cell cycle checkpoints. Changes in *V_m_* are likely to trigger intracellular signaling messengers such as Ca^2+^ in order to drive sustained proliferation.

## Role of *V_m_* in cancer cell migration

**Figure 4:**
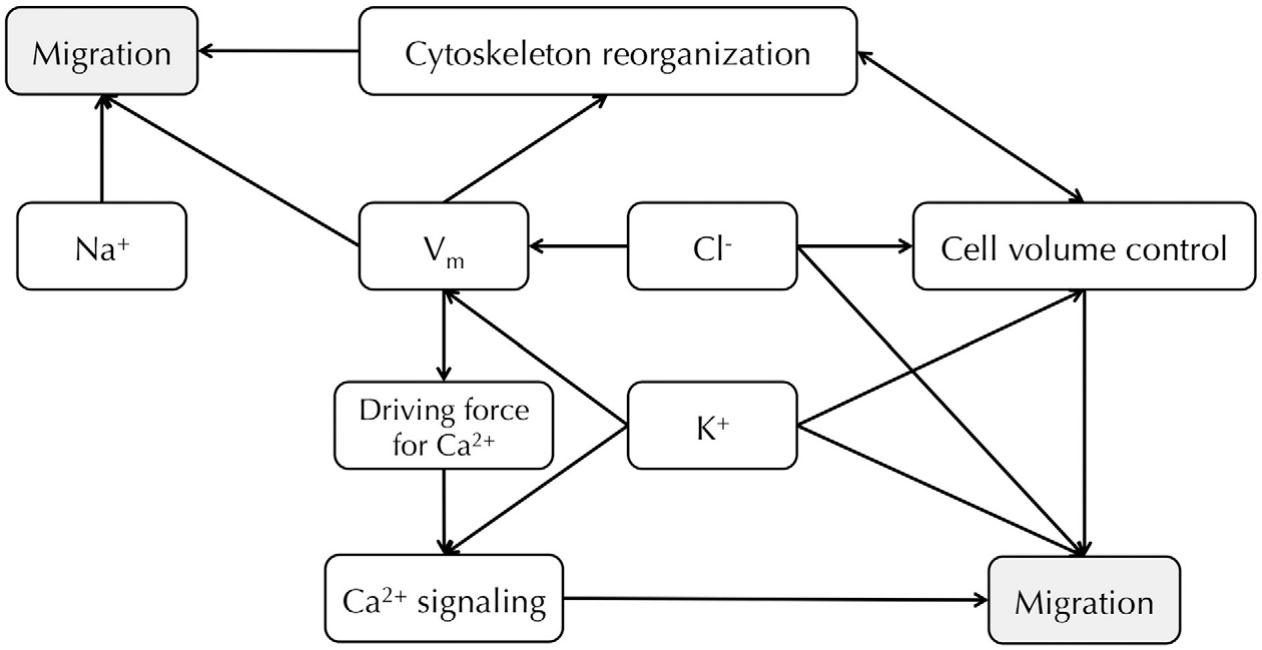
**Relationship between Na^+^, K^+^, Cl^−^ channels and *V_m_* in cancer cell migration**. *V_m_* provides the driving force for Ca^2+^, and downstream Ca^2+^ signaling leads to cell migration (Schwab et al., 2012). *V_m_* also regulates cytoskeleton reorganization (Chifflet et al., 2003, 2004). Cl^−^ and K^+^ channels both contribute to *V_m_* regulation and cell volume control (Soroceanu et al., 1999; Sontheimer, 2008; Habela et al., 2009; Schwab et al., 2012). Inhibiting particular Na^+^, K^+^, and Cl^−^ channels can reduce cancer cell migration (Sontheimer, 2008; Brackenbury, 2012; Schwab et al., 2012).

Metastasis involves loss of adhesion at the primary site, increased migration and invasion, circulation through the vascular/lymphatic systems and growth of secondary tumors at distant sites (Gupta and Massague, 2006; Prevarskaya et al., 2010). Among the various steps in the metastatic cascade, it is well-established that cell migration is tightly controlled by the movement of ions and water [Figure 4; reviewed in depth in Schwab et al. (2007, 2012)]. *V_m_* is regarded as an indirect factor that can affect cell migration, whose main regulatory role might be setting up the electrical driving force for Ca^2+^ (Prevarskaya et al., 2010; Schwab et al., 2012). A hyperpolarized *V_m_* can increase intracellular Ca^2+^ via TRP channels, whereas membrane depolarization could activate voltage-gated Ca^2+^ channels (Schwab et al., 2012). Intracellular Ca^2+^ displays a concentration gradient in migrating cells, with lowest concentration at the leading edge (Brundage et al., 1991). During cell migration, oscillations in Ca^2+^ concentration are observed within microdomains, such that Ca^2+^ flickering is highest in the lamellipodia (Wei et al., 2009). These fluctuations play a role in regulating tractional forces (Lee et al., 1999; Ridley et al., 2003), direction sensing, and cytoskeleton reorganization (Pettit and Fay, 1998). *V_m_* may also affect downstream intracellular signaling cascades that could contribute to cell migration in a Ca^2+^-independent way (Figure 4). For example, in kidney epithelial cells, *V_m_* depolarization induces diphosphorylation of myosin light chain (MLC) without inducing Ca^2+^ signaling, but instead by activating the Rho-Rho kinase (ROK) pathway (Szaszi et al., 2005). In addition, actin filaments undergo reorganization following *V_m_* depolarization in bovine eye endothelial and epithelial cells (Chifflet et al., 2003, 2004), suggesting a functional role for *V_m_* in cytoskeletal reorganization, although it is not clear whether or not Ca^2+^ is involved. Furthermore, applied electrical fields, which would impact on *V_m_*, can enhance motility and galvanotaxis (Djamgoz et al., 2001; Levin, 2003, 2009; Schwab et al., 2012).

A number of Na^+^, K^+^, and Cl^−^ channels, that potentially contribute to the *V_m_*, are directly implicated in cancer cell migration. For example, functional VGSCs have been found in a number of cancer types [reviewed in Brackenbury (2012)], and suppressing VGSCs with siRNA or pharmacological agents inhibits migration and invasion (Roger et al., 2003; Fraser et al., 2005; Brackenbury et al., 2007; House et al., 2010; Yang et al., 2012). In several breast carcinoma/melanoma cell lines, K_*Ca*_2.3, which is responsible for maintaining a hyperpolarized *V_m_*, enhances migration, likely via promotion of intracellular Ca^2+^ signaling (Potier et al., 2006; Chantome et al., 2009). In addition, K_*Ca*_3.1 activity causes a local shrinkage at the rear of migrating MDCK-F cells, therefore supporting retraction at this pole during movement (Schwab et al., 2006). In order to maintain electroneutrality, K^+^ efflux must be accompanied by an anion, and Cl^−^ is the most likely candidate (Schwab et al., 2007, 2012). In agreement with this, Cl^−^ channels, which contribute to the depolarized *V_m_* in glioma cells, enhance migration and invasion by permitting the release of K^+^, Cl^−^, and water at the leading edge, resulting in shrinkage and facilitating movement into tortuous extracellular spaces (Soroceanu et al., 1999; Sontheimer, 2008; Habela et al., 2009; Schwab et al., 2012).

In conclusion, a direct role for *V_m_* in regulating cancer cell migration is much less clear than for proliferation. Given the great variety of ion channels and transporters that are involved in the process of cell migration, the concept of the “transportome” has been proposed (Schwab et al., 2012), which implies that rather than individual ion channels or transporters, it is a complex network of ion translocators that directs the migration and invasion of cells (Figure 4). Further work is required to establish to what extent *V_m_* directly impacts on this network.

## *V_m_* and the differentiation of cancer stem cells

**Figure 5:**
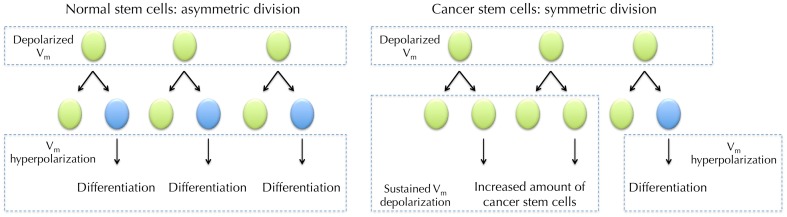
***V_m_* in normal stem cell (SC) differentiation and hypothesized role for *V_m_* in cancer stem cells (CSCs)**. Depolarized *V_m_* is needed during the maintenance of SCs. SC undergoes asymmetric division where it produces one copy of itself and one progeny that later differentiate into mature cells. The maturation requires *V_m_* hyperpolarization (Sundelacruz et al., 2008). However, CSCs frequently undergo symmetric division, in which one CSC divides into two identical CSC progenies (Wicha et al., 2006). Sustained *V_m_* depolarization may help to maintain the increasing CSCs in an undifferentiated state. Proliferation of CSCs then increases cancer malignancy.

Stem cells and cancer cells share similar properties, such as the ability to differentiate and self-renew, increased membrane transporter activity and the ability to migrate and metastasize (Wicha et al., 2006). The cancer stem cell (CSC) hypothesis contains two key concepts: (1) cancers arise from dysregulated transformation of normal tissue stem cells or progenitor cells, and (2) cellular components that display stem cell properties can lead to cancer progression (Wicha et al., 2006). In contrast to normal, regulated asymmetric division of stem cells during tissue homeostasis, where a stem cell produces one copy of itself and one cell that later differentiates into a mature cell, the dysregulation of transformed CSCs during tumorigenesis involves “symmetric division” in which each malign CSC generates two identical daughter cells (giving rise to either proliferation or differentiation), which significantly expands the malign stem cell reservoir (Figure 5) (Liu et al., 2005).

A role for *V_m_* in differentiation of normal stem cells has been previously reported. Studies in quail neural crest cells and a subpopulation of SH-SY5Y cells have demonstrated that stem cells exhibit distinct bioelectrical profiles during development (Arcangeli et al., 1997; Biagiotti et al., 2006; Sundelacruz et al., 2009). In particular, a hyperpolarized *V_m_* is required during stem cell maturation (Sundelacruz et al., 2009). For example, K*_ir_*-induced *V_m_* hyperpolarization is required during human myoblast fusion (Liu et al., 1998). In a genome-wide microarray analysis of depolarization-regulated genes in postnatal mouse cerebellar granule neurons, among 87 depolarization-responsive genes, 22 are developmentally up-regulated and 26 are developmentally down-regulated (Sato et al., 2005). Remarkably, 18 of the 22 (82%) developmentally up-regulated genes coincide with depolarization down-regulated genes, and 20 of 26 (77%) developmentally down-regulated genes with depolarization up-regulated genes (Sato et al., 2005). *V_m_* hyperpolarization is also a functional determinant of human mesenchymal stem cell (hMSC) differentiation. Pharmacologically-induced *V_m_* depolarization suppresses adipogenic and osteogenic differentiation of hMSCs (Sundelacruz et al., 2008). In addition, depolarization reduces the differentiated phenotype of hMSC-derived cells and improves their ability to transdifferentiate, without fully restoring a stem cell-like genetic profile (Sundelacruz et al., 2013). Taken together, these data suggest that *V_m_* depolarization may maintain cells in an undifferentiated stage at the gene expression level. Therefore, it is not unreasonable to postulate that depolarized *V_m_* may also help maintain a population of undifferentiated CSCs (Figure 5). This possibility would raise additional, related questions: does a more depolarized *V_m_* promote the proliferation of CSCs? Does *V_m_* affect the pattern of symmetric vs. asymmetric division? Further work is required to investigate these possibilities.

## Clinical implications

Given that the fluctuation of *V_m_* can functionally regulate tumorigenesis, differentiation, and promote cancer progression, it may serve as a potential marker for tumor detection and treatment, with prognostic value. For example, bioelectrical impedance analysis, which determines tissue electrical properties, has shown promise as a prognostic indicator to monitor cancer progression (Gupta et al., 2004a,b); , and recently, the development of non-invasive, voltage-sensitive optical probes provides a potential approach for *in vivo V_m_* measurement (Adams and Levin, 2012; Chernet and Levin, 2013). Considering the vast array of therapeutic drugs that target ion channels (Sontheimer, 2008; Stuhmer and Pardo, 2010; D'amico et al., 2013; Djamgoz and Onkal, 2013), modulating the *V_m_* of malign tissues by adjusting the activities of varies ion channels/transporters may provide a convenient clinical approach.

### Conflict of interest statement

The authors declare that the research was conducted in the absence of any commercial or financial relationships that could be construed as a potential conflict of interest.
